# Ultrasonography to detect cardiovascular damage in children with essential hypertension

**DOI:** 10.1186/s12947-021-00257-y

**Published:** 2021-07-21

**Authors:** Wei Liu, Cui Hou, Miao Hou, Qiu-Qin Xu, Hui Wang, Pei-Pei Gu, Ling Sun, Hai-Tao Lv, Yue-Yue Ding

**Affiliations:** Cardiology Department, Children’s Hospital of Soochow University, Suzhou City, Jiangsu Province 215025 China

**Keywords:** Hypertension, Echocardiography, Cardiovascular damage, Children, Noninvasively

## Abstract

**Background:**

Essential hypertension in adults may begin in childhood. The damages to the heart and blood vessels in children with essential hypertension are hidden and difficult to detect. We noninvasively examined changes in cardiovascular structure and function in children with hypertension at early stage using ultrasonography.

**Methods:**

All patients with essential hypertension admitted from March 2020 to May 2021 were classified into simple hypertension (group 1, n = 34) and hypertension co-existing with obesity (group 2, n = 11) isolation. Meanwhile 32 healthy children were detected as control heathly group (group 3). We used pulse-wave Doppler to measure carotid–femoral pulse wave velocity (cfPWV), intimal–medial thickness (cIMT) and distensibility of carotid artery (CD). Cardiac structure and function (left atrial diameter [LAD], left ventricular mass [LVM], LVM index [LVMI], relative wall thicknes [RWT], end-diastolic left ventricular internal diameter [LVIDd], diastolic interventricular septum thickness [IVSd], diastolic left ventricular posterior wall thickness [LVPWd], root diameter of aorta [AO], E peak, A peak, E' peak, A' peak, E/E' ratio, and E/A ratio) were measured by echocardiography.

**Results:**

The cfPWV of children in group 1 and group 2 were significantly higher than healthy children in group 3. Significant differences were observed in LVM, LVMI, RWT, LVIDd, IVSd, LVPWd, LAD, A peak, E' peak, A' peak, and E/E’ among three groups.

**Conclusion:**

Children and adolescents with essential hypertension demonstrate target organ damages in the heart and blood vessels.

## Introduction

Essential hypertension (HTN) in adults may begin in childhood (the so-called trajectory phenomenon [1]. The mortality and morbidity of hypertension in children and adolescents are closely related to cardiovascular disease in adults. However, hypertension has a low incidence of cardiovascular events in children and adolescents. Therefore, it is of great significance to study whether hypertension causes structural and functional damage to the heart and blood vessels in childhood and adolescence, so that early target organ damages can amply proves to cardiovascular events in adulthood.

The diagnostic criteria for HTN in children are not uniform and are generally considered higher than percentile values for blood pressure in this age group. In 2017, Jie Mi et al. [2] updated the blood pressure standards for Chinese children issued in 2010. They also developed blood pressure reference standards for children based on sex, age, and height [2] In recent years, the prevalence of hypertension among Chinese children and adolescents has been on the rise. Hypertension usually has no obvious clinical manifestations. Thus, people often lack vigilance, leading to delayed treatment. Many children and adolescents with abnormal blood pressure continue into adulthood. Thus, hypertension in children and adolescents develops into adult hypertension, causing heart, brain, kidney and other target organ damage, as well as atherosclerosis.

Over time, essential hypertension leads from structural changes in heart to systolic and diastolic dysfunctions eventually culminate in heart failure. Although heart failure is uncommon in children and adolescents with hypertension, it is important to assess the presence of early changes in diastolic and systolic function. Hypertension in adults is often associated with the heart and other target organs. However, studies on target organ damage in children with essential hypertension are lacking.

Hypertension can also lead to changes in vascular structure and function. There are multiple models available to assess vascular structure and function, which are divided into three categories: vascular structure, arterial stiffness, and endothelial function. Pulse wave velocity (PWV) is the most commonly measured non-imaging parameter to assess arterial stiffness. Carotid intimal–medial thickness (cIMT) is the primary index for measuring vascular structure.

This study aimed to noninvasively evaluate changes in cardiovascular structure and function in children with hypertension by ultrasonography to verified the target organ damages.

## Materials and methods

### Subjects

We continously retrospectively studied 45 children (34 simple hypertensive in group 1, 11 hypertensive co-existing with obesity in group 2) with newly diagnosed essential hypertension admitted in the cardiology department in the Children’s Hospital of Soochow University from March 2020 to May 2021, and 32 healthy children matched with age and sex were recruited from the community-based population as the control healthy group 3. Clinical parameters (Table 1), including age, gender, body mass index (BMI; kg/m2), blood pressure (BP) and biochemical data on lipids were obtained in all children. Hypertension was diagnosed on systolic and/or diastolic pressure ≥ 95th percentile for sex, age, and height according to the reference value of the Chinese Child Blood Pressure References Collaborative Group_。_[2] Obesity was defined as BMI > 95th percentile for age, sex, and height.

**Table 1:** The Clinical Characteristics of Children in Three Groups

variables	Hypertensive (n = 34)	hypertensive + obese (n = 11)	Control (n = 32)	*p* value
Sex (male/female)	25/9	6/5	17/14	0.202
Age (years)	11.8 ± 2.6	11.5 ± 3.0	10.9 ± 2.5	0.365
Weight (kg)	59.5 ± 17.6	77.0 ± 22.1	43.0 ± 14.1	0.000
Height (cm)	160.2 ± 17.7	156.9 ± 17.2	152.2 ± 18.2	0.192
BMI (kg/m^2^)	22.4 ± 3.9	30.6 ± 4.9	18.0 ± 2.8	0.000
SBP(mmHg)	144.4 ± 16.3*	144.6 ± 14.0 *	106.5 ± 11.3	0.000
DBP(mmHg)	85.6 ± 14.1 *	94.6 ± 13.8^**#**^	65.9 ± 6.0	0.000
PP(mmHg)	58.8 ± 11.9 *	50.0 ± 15.4^**#**^	40.9 ± 10.6	0.000
TG(mmol/L)	1.3 ± 0.7	1.6 ± 0.7	1.3 ± 0.5	0.361
TC(mmol/L)	4.3 ± 1.0	5.0 ± 0.8	4.2 ± 0.7	0.103
HDL(mmol/L)	1.2 ± 0.3	1.2 ± 0.1	1.4 ± 0.3	0.210
LDL(mmol/L)	2.6 ± 0.9	3.4 ± 0.6	2.8 ± 0.4	0.091
drug therapy for hypertension	No drugs	No drugs	-	-

*Abbreviations*: *BMI* Body mass index, *SBP* Systolic blood pressure, *DBP* Diastolic blood pressure, *PP* Pulse pressure, *TG* Triglycerides, *TC* Total cholesterol, *HDL* High-density lipoprotein cholesterol, *LDL* Low-density lipoprotein cholesterol. * and ^#^ indicates there is statistical difference compared with the healthy group and *P* < 0.05, respectively

Among the 45 hypertension patients, 8 patients were admitted with obese and 5 children were admitted with dizziness and chest tightness as the chief complaint. 31 patients were admitted with high blood pressure on routine physical examination, and 1 patient was admitted to hospital for digestive tract foreign body. All the children were newly diagnosed with essential hypertension and had not been treated with drugs. They were excluded secondary hypertension, cardiomyopathy, and valvular heart disease at the time of admission to hospital, meanwhile subjects with hyperthyroidism or diabetes mellitus were also excluded.

## Method

### Blood pressure measurement

Irritant drugs and food were not permitted before measurement. Patients were required to sit for 5–10 min in a quiet environment, with the cubital fossa and heart at the same level. A standard clinical cuff sphygmomanometer was used to measure blood pressure in the right upper arm. The chest part of the membrane stethoscope was placed on the medial side of the cubital fossa for brachial artery pulsation (2 cm above the elbow fossa). We took the K1 tone as the standard for systolic blood pressure and the K5 tone as the standard for diastolic blood pressure. We performed measurements twice continuously and averaged the two measurements. The interval between each measurement was 3 min. If the difference between the first two readings was > 5 mmHg, we used the average value after obtaining a third reading. The first diagnosis of hypertension was verified by a third examination.

### Echocardiography

#### Measurement of cardiac structure

Echocardiography was performed using a Philips EPIQ7C color Doppler ultrasound machine ( Koninklijke Philips Ultrasound Inc., Netherland), equipped with S8-3, S5-1 and L12-5 probes ranging from low to high frequency (3–6 MHz, 1.6–3.2 MHz and 4.4–8.8 MHz, respectively). According to American Society of Echocardiography guidelines for pediatric echocardiogram from 2006 [3], we measured diastolic left ventricular internal diameter (LVIDd), diastolic interventricular septum thickness (IVSd), and diastolic left ventricular posterior wall thickness (LVPWd), diameter of aortic root (AO) and left atrial diameter (LAD).

#### Measurement of cardiac diastolical function

The Doppler spectrum of the mitral valve pulse was recorded in an apical four-chamber view. The peak velocity of the filling peak in the early diastolic period (E) and the peak velocity of the filling peak in the late diastolic period (A) were measured, and the E/A ratio was calculated. Three cardiac cycles were measured, and the average value was used. We used the Tissue Doppler Imaging technique to measure the velocity of the mitral annulus (E') and calculated the E/E' value by combining this with the velocity of early diastolic blood flow (E) through the mitral valve orifice. This study defined left ventricular diastolic dysfunction as an E/E' > 15 or an E/A < 1.0.

#### Left ventricular remodeling

The calculations of left ventricular mass (LVM), LVM index (LVMI), and relative wall thickness (RWT) were based on Devereux formulas:

$${\text{LVM }}\left( {\text{g}} \right) \, = \, 0.{8}0 \, \times [{1}.0{4 } \times \, \left( {{\text{IVSd }} + {\text{ LVPWd }} + {\text{ LVIDd}}^{{3}} - {\text{ LVIDd}}^{{3}} } \right)] + 0.{6}.$$

$${\text{LVMI }}\left( {{\text{g}}/{\text{m}}^{{{2}.{7}}} } \right) \, = {\text{ LVM}}/{\text{height}}^{{{2}.{7}}} .$$

$${\text{RWT }} = \, \left( {{\text{IVSd }} + {\text{ LVPWd}}} \right)/{\text{LVIDd}}.$$

Three cardiac cycles were measured, and the average value was taken. The criterion for left ventricular hypertrophy (LVH) was a LVMI > 38.6 g/m ^2.7^ in children. An RWT > 0.41 was considered abnormal [4]. According to echocardiography, there are four possible left ventricular configurations: concentric hypertrophy, eccentric hypertrophy, concentric remodeling, and a normal configuration (as judged by LVMI and RWT).

### Ultrasonography

#### Measurement of arterial stiffness

**Fig. 1:**
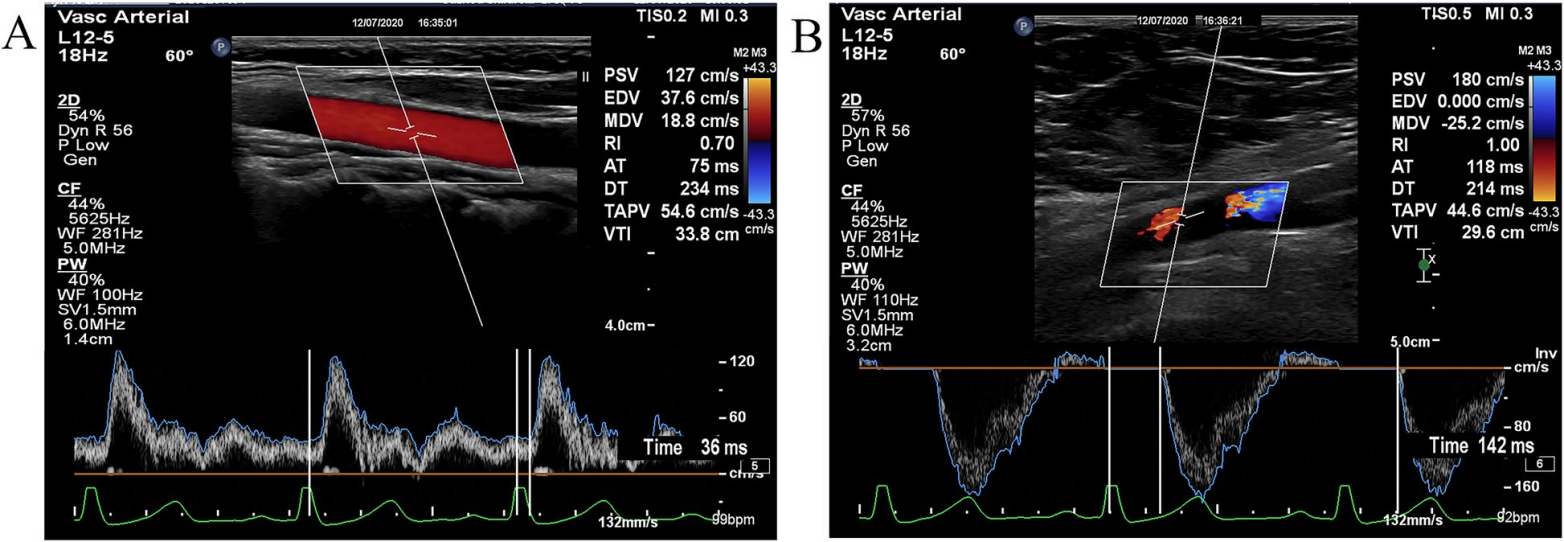
The Measurement Method of Carotid-femoral PWV. **A** indicate the time measurement of carotid artery from the R wave of QRS to the foot of the waveform. **B** indicate the time measurement of femoral artery from the R wave of QRS to the foot of the waveform

Carotid–femoral PWV (cfPWV) was calculated as the ratio of the travel distance (D) to carotid–femoral pulse transit time (T). The path length of the direct carotid-femoral distance segment (D) was estimated based on the surface distance (Ds) from the carotid arteral measurement point to the femoral measuremental point using the formula: D = Ds × 0.8 (a correction of the distance for a fixed factor of 0.8 was applicated jn order to encount the simultaneously traveling of the pulse wave from aorta to carotid and to femoral arteries) [5]. The so-called transit time (T) was the time of travel from carotid artery to femoral artery of the wave over the distance. cfPWV was calculated as follows: cfPWV = D (meters) / T (seconds). Each participant was examined in supine position and the head slightly back while electrocardiography was connected synchronously. The carotid artery point was located at a distance of 1.0–2.0 cm to the carotid bifurcation and marked on the body surface. The process was repeated at the femoral artery point in the groin position with three repeated measurements. We use a caliper to measure the time from the ECG R wave peak to the start of the waveform as the wave transmission time (Fig. 1).Two measurements were obtained in each patient and the mean was used for the analysis. The acquisitions were all performed by the same operator in a double-blind condition to exclude inter-group differences.

#### Measurement of vascular structure

Patients assumed a supine position to expose the neck. Ultrasonography of the carotid artery lumen was performed, and cIMT of the anterior and posterior walls was measured. At 1–2 cm below the bifurcation plane, the thickness of the internal–medial carotid membrane were automatically measured by the built-in software package.

Carotid distensibility (CD) can be measured by acquiring carotid artery diameter in end diastole and the systo-diastolic change also called distension or the diameter changes of carotid artery (∆D). The systolic and diastolic diameter of carotid artery were manually measured, then calculated ∆D. After that CD is calculated according to the reneman formula:

$$\begin{array}{*{20}c} {\Delta {\text{D}} = {\text{Dd }} - {\text{Ds}}} \\ {\Delta {\text{D}}\% \, = \, \left( {{\text{Dd }} - {\text{ Ds}}} \right) \, /{\text{ Dd}} \times {1}00\% } \\ {{\text{CD }} = \, [({2}\Delta {\text{D}} \times {\text{Dd}}) \, + \Delta {\text{D}}^{{2}} ] \, /{\text{ PP}} \times {\text{Dd}}^{{2}} .} \\ \end{array}$$

where Dd is the end diastolic diameter of the vessel, Ds is the systolic diameter of the carotid artery. PP is the central pulse pressure [6].

### Statistical analysis

We used SPSS 25.0 software (SPSS Inc., Chicago, IL) to perform statistical processing of the data. Measurement data are expressed as mean ± standard deviation. As we were primarily interested in comparing both disease groups (hypertension and hypertension + obesity) with the healthy control group, we performed one-way analysis of variance (ANOVA), where the disease groups were considered mutually exclusive. All results are presented from the one-way ANOVA. A *P* value of < 0.05 was considered statistically significant.

## Results

The clinical characteristics and biochemical parameters of hypertension and control groups are shown in Table 1. There was no significant difference in age, sex, height, triglycerides (TG), total cholesterol (TC), high-density lipoprotein cholesterol (HDL) and low-density lipoprotein cholesterol (LDL) among the three groups (*P* > 0.05). There were significant differences in body weight, BMI, systolic blood pressure (SBP), diastolic blood pressure (DBP) and pulse pressure (PP) in group 1 and group 2 compared with group 3 (*P* < 0.05).

There were significant differences in LVM, LVMI, RWT, LVIDd, IVSd, LVPWd, LAD, A peak, E' peak, A' peak and E/E' among the three groups (P < 0.05). LVM, RWT, LVIDd, LVPWd, LAD and E/E’ in group 1 and group 2 were significantly higher than the healthy control group. The two hypertension groups were not significantly different from each other. IVSd and LVMI were significantly different among the three groups by S–N-K test. A peak and A' peak were significantly higher in group 1 compared with group 2 and group 3 in a post-hoc analysis. Moreover, E’ peak was significantly lower in group 2 compared with group 1 and group 3. However, there was no difference in AO, E peak and E/A ratio among the three groups (Table 2).

**Table 2:** Comparison of Cardiac Structure and Diastolic Function in Three Groups

variables	Hypertensive (n = 34)	Hypertensive + Obese (n = 11)	Control (n = 32)	*p* value
LVM (g)	128.1 ± 37.8 *	142.9 ± 51.0 *	82.5 ± 28.1	0.000
LVMI (g/m^2.7^)	34.1 ± 7.0 *	43.3 ± 11.6^**#**^	26.2 ± 6.1	0.000
RWT	0.36 ± 0.058 *	0.39 ± 0.057 *	0.30 ± 0.039	0.000
LVIDd (mm)	46.3 ± 4.6 *	46.1 ± 6.1 *	42.4 ± 4.7	0.006
IVSd (mm)	8.2 ± 1.6 *	9.3 ± 1.0^**#**^	6.6 ± 1.1	0.000
LVPWd (mm)	8.1 ± 1.4 *	8.4 ± 1.9 *	6.3 ± 1.2	0.000
AO (mm)	22.7 ± 3.0	22.1 ± 2.5	20.1 ± 4.1	0.087
LAD (mm)	28.2 ± 3.8 *	28.2 ± 4.3 *	23.5 ± 3.4	0.000
E peak (m/s)	111.2 ± 16.1	103.9 ± 17.1	106.2 ± 17.1	0.352
A peak (m/s)	68.3 ± 20.6 *	55.6 ± 17.2	51.2 ± 11.9	0.001
E' peak (cm/s)	12.6 ± 2.1	10.5 ± 1.4 *	13.9 ± 1.8	0.000
A' peak (cm/s)	8.1 ± 1.7 *	6.4 ± 1.3	5.9 ± 1.5	0.000
E/E'	9.0 ± 1.6 *	10.0 ± 1.6 *	7.7 ± 1.1	0.000
E/A	1.7 ± 0.5	2.2 ± 1.1	2.2 ± 1.0	0.067

*Abbreviations*: *LVM* Left ventricular mass, *LVMI* Left ventricular mass index, *RWT* Relative wall thickness, *LVIDd* Left ventricular internal diameter at end-diastole, *IVSd* Diastolic interventricular septum thickness, *LVPWd* Diastolic left ventricular posterior wall thickness, *AO* diameter of aortic root, *LAD* Left atrial diameter, *E peak* E peak of mitral valve pulse by doppler spectrum, *A peak* A peak of mitral valve pulse by doppler spectrum, *E’ peak* E’ peak of mitral valve pulse by tissue doppler imaging, *A’ peak*: A’ peak of mitral valve pulse by tissue doppler imaging, *E/E’* E peak/E’ peak, *E/A* E peak/A peak. * and ^#^ indicates there is statistical difference compared with the healthy group and *P* < 0.05, respectively

A 0.31 cut-off value of RWT had 85.7% sensitivity and 77.4% specificity for hypertension patients. A cut-off value of cfPWV is 4.55 m/s, and this value had 88.9% sensitivity and 53.6% specificity for hypertension patients. 2 of 11 children in HTN + Obese group were concentric hypertrophy (2/11, 18.1%), 2 children were eccentric hypertrophy (2/11, 18.1%) and 1 child were concentric remodeling (1/11, 9.0%), respectively. Of the 34 HTN patients, 2, 6 and 5 children had concentric hypertrophy(2/34, 5.9%), eccentric hypertrophy(6/34, 17.6%) and concentric remodeling(5/34, 14.7%), respectively.

**Fig. 2:**
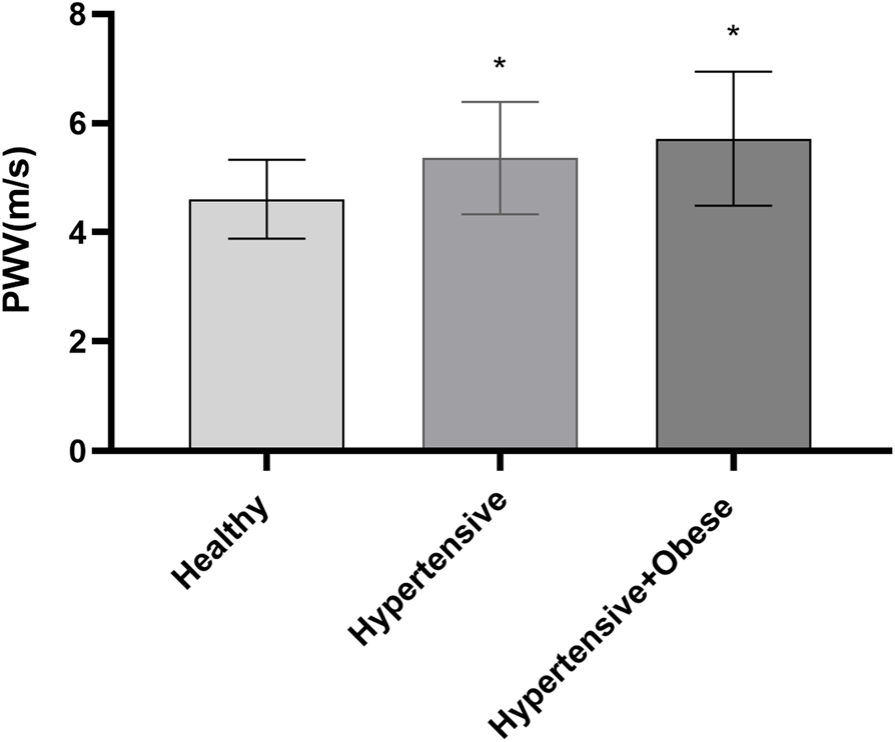
The Differences of PWV in The Three Groups. Column bar graph shows the differences of PWV among the Healthy group, the Hypertensive group, and the Hypertensive and Obese group. * indicate *P* value < 0.01

There were significant differences in cfPWV among the three groups. cfPWV was significantly higher in both hypertension groups in comparison with the healthy control group (Fig. 2). There were no significant differences in cIMT, ∆D% and CD among three groups as shown in Table 3.

**Table 3:** Vascular Structure and Function Difference in Three Groups

variables	Hypertensive (n = 34)	hypertensive + obese (n = 11)	Control (n = 32)	*p* value
IMT (mm)	0.46 ± 0.05	0.46 ± 0.05	0.44 ± 0.05	0.355
PWV (m/s)	5.4 ± 1.0 *	5.7 ± 1.2 *	4.6 ± 0.7	0.001
∆D%	16.8 ± 4.3	15.4 ± 3.7	16.1 ± 4.6	0.640
CD	9.6 ± 5.6	13.6 ± 7.3	12.0 ± 7.4	0.177

*Abbreviations*: *IMT* intima-media thickness, *PWV* carotid-femoral pulse wave velocity; ∆D%: Rates of diameter changes of carotid artery(%); CD: Carotid distensibility. *indicates there is statistical difference compared with the healthy group and *P* < 0.05

## Discussion

In 2017, with the blood pressure standards for Chinese children by sex and age, and height developed, hypertension in children and adolescents aroused great concern in pediatrics. It is the most widely used diagnostic criteria. Our definition of hypertension is based on this criterion, and our study found the structural changes in heart, cardiac diastolic dysfunction and vascular stiffiness.

There were significant differences in LVM, LVMI, RWT, LVIDd, IVSd, LVPWd, LAD in hypertensive children. An increase in afterload caused by high blood pressure leads to an increase in ventricular wall stress and eventually changes in myocardial structure, which is called left ventricular hypertrophy or remodeling. In patients with continuous hypertension in the systemic circulation, cardiac afterload increases, resulting in an increase in left ventricular end-systolic residual blood volume and diastolic volume. This in turn results in left ventricular compensatory hypertrophy, a decrease in myocardial elasticity, and an increase in left ventricular volume. Then an increasing in left atrial residual blood volume at the end of diastole and gradual left atrial enlargement maintain an atrioventricular pressure difference and excessive cardiac output [7]. Left ventricular remodeling refers to a series of changes in the shape, structure, and function of the heart as hypertension progresses.

High blood pressure leads to an increase in myocardial mass, which is reflected by an increase in LVIDd, IVSd, LVPWd of cardic morphology and structure. An increase in LVM is a manifestation of left ventricular hypertrophy (LVH) and an independent risk factor for LVH [8]. Using the Devereux formula to calculate LVM and correcting LVM for height to obtain LVMI is a common method to judge whether or not LVH exists. Our study confirmed that LVMI is significantly higher in children with hypertension especially obesity than healthy children just like the other published litertures.

RWT is also an important indicator of cardiovascular remodeling in hypertension. Although the RWT of hypertensive groups were higher than that of the control group, their values were all within the normal range in our study. A 0.31 cut-off value had 85.7% sensitivity and 77.4% specificity for hypertension patients while a 0.41 cut-off value only had 21.4% sensitivity and 100% specificity. We should establish a new cut-off value for RWT in children with hypertension.

Among children with 45 hypertension, 12 (12/45, 26.7%) ones had LVH and 6 children(13.3%) had left ventricular remodeling. The incidence of LVH was consistent with the 27% reported by Sorof [9], so LVMI and RWT can be used as clinical evidence of target organ damage in children with hypertension. Although 60% of patients in this study showed a normal configuration, the rate of LVH was significantly higher compared with healthy controls. Echocardiography to diagnose LVH and remodeling, to follow up in screening and to manage hypertension is a viable strategy.

Studies had shown a positive correlation between high blood pressure and obesity in children and adolescents [10, 11]. The prevalence of hypertension in children with obesity is > 10-times that of children with a normal BMI. Studies had confirmed that the earlier obesity occurs, the longer its duration, and the greater the likelihood of hypertension. In our study, obese children with hypertension had higher diastolic blood pressure and more impaired left ventricular diastolic function. And other indicators were consistent with those of children with hypertension. The ratio of left ventricular hypertrophy and remodeling in group 2 was higher than that in group 1. Both hypertension and obesity are considered independent risk factors for LVMI. To avoid errors in estimating the effects of being overweight LVMI was better than LVM in distinguishing the damage to the heart caused by hypertension.

In addition to changes in left ventricular structure, changes in left atrial structure and function have been confirmed as early pathological changes in patients with hypertension [12]. In our study, there was significant difference in LAD between the three groups. The result shows that children of hypertension groups demonstrates left atrial enlargement compared with healthy children, which is consistent with the results of Tsai [13]. Keller et al*.* [14] also found that even if echocardiography showed no myocardial hypertrophy, LAD would increase significantly. It is possible that left atrial enlargement exists before LVH, because the atrial muscle fibers are smaller and shorter than ventricular muscle fibers, so they are also more sensitive to pressure.

Previous study defined left ventricular diastolic dysfunction as an E/E' of > 15 or an E/A of < 1.0. We compared E/E' ratio between the three groups and found that although E/E' in children with hypertension was < 15, children in these groups had a significantly increased E/E' compared with the healthy group. This indicated that left ventricular diastolic function decreases in the groups with hypertension. We found that the E' peak of the ventricular septum in group 2 was significantly decreased, and A' peak of the ventricular septum and A peak of mitral valve were higher in group 1 than the other two groups. These results proved that increased left atrial pressure and impaired left atrial function were common in children with hypertension. There was no significant difference in E/A ratio between the three groups but the E/E' in children with hypertension whatever obese or not was significantly increased. E/A probably is less sensitive than E/E’ as previously reported literatures [15, 16]..

In addition to the above cardiac damage, hypertension can also cause changes in vascular structure and function. Arterial stiffness or arterial compliance can be measured as PWV which was the speed of pulse wave transmitted across the length of the arterial tree. A higher PWV indicates a stiffer blood vessel, contributing to increased afterload and subsequent cardiac remodeling [17]. In adults, a higher PWV predicts the risk of cardiovascular disease events, such as stroke, ischemic heart disease, and hypertension [18, 19]. A meta-analysis of > 15,000 subjects confirmed that an increase in PWV of 1 m/s results in an increase of 14% in the risk of cardiovascular events after adjustment for age, sex, and cardiovascular risk factors, and an increase of 15% in cardiovascular mortality [20]. There are a few studies on PWV on adolescent HTN. Kulsum-Mecci N et al. found adolescent aged 4 to 18 years of obesity and HTN both significantly and independently increased PWV, while PWV increased with age but did not differ by race or sex [21].

The predictors of PWV were age, SBP, heart rate, BMI, antihypertensive treatment and drug classes, not related to race, gender, smoking, dyslipidaemia, diabetes, kidney disease, or genetic factors. Two factors were identified as being responsible for accelerated progression of PWV after correction for HR: age and BP values [22]. However, in our study, there was no difference in biochemical data on blood lipids, no use of antihypertensive drugs, and no difference in age and sex. Therefore, most of the related predictors could be excluded and the influence of BP could be considered importantly[23]. Meanwhile, in our study, a relative increasing in cfPWV in HTN with or without obesity indicated that HTN had a greater impact on vascular stiffness than obesity. Studies by some groups had shown an increase in PWV (a decrease in arterial compliance) with obesity alone [24, 25]. But one limitation of our study was missing parameters of simple obesity group.

Doppler methods to assess cfPWV is feasible. It is not the preferred one compared to the automatic one using tonometey or pressure sensor, but it’s a good replacement to assess cfPWV when you don’t have the automatic machine. Although Doppler-PWV measurements determined more evaluation error than ones of applanation tonometry, piezoelectric mechano-transducer or cuff-based oscillometry, Doppler-PWV exactly showed close correlation with invasive assessment as well. Styczynski G. et al. [26] found that mean invasive PWV was 9.38 m/sec and mean echo-PWV was 9.51 m/sec(P = 0.78), the Pearson’s correlation coefficient between methods was 0.93 (P < 0.0001), a Bland–Altman plot revealed a mean difference between invasive PWV and echo-PWV of 0.13 ± 0.79 m/sec. Doppler-PWV is a reliable method of PWV measurement. Wider implementation of the Doppler-PWV method for the evaluation of aortic wall stiffness can further expand the clinical and scientific utility of echocardiography [27].

In our study, absolute cfPWV values in the three groups were much lower than the threshold for severe cardiovascular events in adults (10 m/s), but both HTN groups had significantly higher values than the healthy children. We found that 4.55 m/s cut-off value of cfPWV had 88.9% sensitivity and 53.6% specificity for hypertension patients. Although 10 m/s cut-off value had gained independent predictive value for fatal and nonfatal cardiovascular events in hypertensive adult patients, this threshold did not distinguish HTN let alone stratify them in child and adolescents. We need more studies of vascular PWV in children to further determine the cut-off value and reclassify intermediate risk patients into higher or lower target organ damage risk.

There were no significant difference in carotid IMT and CD between the three groups of children, probably because our sample size was too small to stratified analysis based on age, weight and height. Carotid ultrasound measurement of IMT is the most commonly used assessment of vascular structure, since it is considered to reflect overall atherosclerotic burden. Currently, many machines had built-in software that can automatically measure IMT to reduce manual measurement error. Kollias et al. [28] studied the relationship between ambulatory BP and cIMT. The mean cIMT in children with a higher BP was 0.03 mm larger than those with a normal BP in a meta-analysis. Day TG et al. [29] have found that a higher BP is associated with a higher cIMT, even after adjusting for cardiovascular risk factors, but they did not have a clear diagnostic criteria of BP for the observed effect. It should be noted that cIMT measurements in children may vary greatly between imaging protocols and machines, but absolute values are much lower than those associated with severe cardiovascular events in adults (usually > 1.0 mm).

Carotid distensibility is probably the most standardized and used carotid arterial stiffness index. Measuring CD means quantify changes in arterial diameter in response to BP changes from diastole to systole. Like cIMT distensibility has also been related to all cause mortality, cardiovascular morbidity and mortality and the presence and severity of cardiovascular diseases in adults [30]. It represents an evaluation of functional abnormalities before structural modification began. Elaine M et al. [31] found that prehypertensive youth had increasing arterial stiffness and a graded increase in cIMT. But our study found no differences in CD and cIMT among the three groups. The possible reasons were that the sample size was small, the inner diameter changes of carotid artery were measured manually not by automatic instrument, and the pulse pressure measured was non-central method et al.

There are some limitations of this study that should be noted. First, this was a retrospective study, thus selection bias was inevitable. Second, the sample size was small, and the regional distribution was uneven, and because of the small sample size of children with obesity, we did not evaluate the effects of obesity on cardiovascular structure and function alone. Finally, a coefficient of variation of the mean value of the intra-session within- and between-operator variability respectively was missing and there was no agreement data between the Doppler technique and tonometry or piezoelectric mechano-transducer technique, as well as intra- / inter- reproducibility for our center when assessing cfPWV. In view of these limitations, follow-up studies should expand the sample size and increase the analysis of cardiovascular effects in individuals with obesity. Our future research directions include longitudinal studies to assess changes in cfPWV over time in healthy children and children with hypertension and obesity. We can do further consistency study between ultrasonographic and automatic tonometey methods.

In conclusion, although essential hypertension has no obvious clinical symptoms, Doppler ultrasonography showed that the heart and blood vessels of children with hypertension undergo structural and functional changes. Our data provide fundamental support for the argument that hypertension is having an effect on target organ damage of cardiovascular system in adolescents and young adults.

## Conclusions

Doppler ultrasonography shows that the heart and blood vessels of children with hypertension undergo structural and functional target organ damages.

## Data Availability

The data and material in the current study are available from the corresponding author on reasonable request.
